# The IL-6 -634C/G polymorphism: a candidate genetic marker for the prediction of idiopathic recurrent pregnancy loss 

**Published:** 2016-02

**Authors:** Zarnegar Rasti, Mahboobeh Nasiri, Leila Kohan

**Affiliations:** 1 *Department of Biology, Islamic Azad University, Arsanjan Branch, Arsanjan, Iran.*; 2 *Young Researchers and Elite Club, Islamic Azad University, Arsanjan Branch, Arsanjan, Iran.*

**Keywords:** *Polymorphism*, *Interleukin-6*, *Recurrent pregnancy loss*

## Abstract

**Background:**

Recurrent pregnancy loss (RPL) is defined as two or more miscarriages before the 20^th^ week of gestation and its etiology is unknown in 50% of the cases. Interleukin 6 is an immune mediator, plays a regulatory role in embryo implantation and placental development.

**Objective:**

The purpose was to assess the association between IL-6 -634C/G polymorphism and, susceptibility to idiopathic RPL for the first time in Iran.

**Materials and Methods:**

In total 121 women with RPL and 121 healthy women as control group were enrolled in this case-control study. This study was performed from August 2013 to October 2014 in the Molecular Genetics Laboratory of Arsanjan University. Candidate polymorphism was evaluated by PCR-RFLP method on extracted genomic DNA. Data was analyzed using the statistical SPSS package.

**Results:**

Our results showed an increased risk of RPL in patients with GG + GC genotype (OR=5.1, 95%CI: 1.04-25.3, p=0.04) in comparison to CC genotype. The frequency of mutant allele G in patients and controls was 0.75 and 0.66 respectively. The mutant allele G predisposes women to miscarriage 1.5 times greater than controls (OR=1.5, 95%CI: 1.03-2.27, p=0.036). The mean number of live births in RPL women (1.3±2.3) was significantly lower compared to control women (4.8±2.3).

**Conclusion:**

This study indicated that the promoter polymorphism (-634C/G) of the IL-6 gene has likely inﬂuence on individual susceptibility to RPL.

## Introduction

Recurrent pregnancy loss (RPL) is a serious complication of pregnancy with the frequency of 1-5% among fertile couples trying to conceive (1). RPL is defined as “the occurrence of 2-3 or more consecutive pregnancy loss prior to 20th week of gestation” in reproductive age (2). RPL is a genetically heterogeneous condition resulted from both maternal and embryonic regulating factors (3, 4). Along with some known causes such as chromosomal abnormalities, metabolic disorders, anatomical anomalies of uterus and immunological factors, the etiology in approximately 50% of the RPL cases remains unknown (idiopathic RPL) which might be explained by immunological factors (5, 6).

Since, fetus expresses the antigen inherited from both parents, survival of semi- allograft fetus till the term, is one of most challenging pregnancy related concepts (7, 8). The maternal immune system plays its critical role in successful pregnancy by controlling fertilization, implantation, progression and maintenance of pregnancy products (9-11). Therefore, investigation of immune cells, immune mediators/cytokines in the peripheral blood of mother and their coding genes would open the new window on understanding at least maternal causes of RPL. Cytokines are cell signaling proteins, mainly secreted by immune cells and mediated cell to cell communication. They are also involved in immunological, inflammatory and infectious diseases (12). 

They act beyond immune system in several developmental processes during embryogenesis, all steps of reproduction and then play a critical role in pregnancy outcome (13). Depending on inflammatory reactions, cytokines sub-divided into pro-inflammatory and anti-inflammatory, which are produced by T helper-1 (Th-1) and Th-2 cells, respectively. Pro-inflammatory cytokines inhibit trophoblast growth and differentiation, while anti-inflammatory cytokines promote embryonic development and placentation. On the other hand, these anti-inflammatory cytokines antagonistically control the action of Th-1 dependant cytokines (14-16). 

In this regard, Th-2 type immunity is believed to contribute to normal and successful pregnancy and Th-1 type immunity have been shown to be associated with pregnancy failure and may be with idiopathic recurrent miscarriage (IRM) (16). However, successful pregnancy is dependent on maintaining a fine balance between Th-1 and Th-2 immunity as well as their specific secretary cytokines (17). Interleukin (IL)-6 is a multifunctional pro- and anti-inflammatory 21-28 KDa glycoprotein, produced by different type of cells, mainly T-lymphocytes, macrophages and monocytes (12, 18). 

The gene encoded for IL-6 is mapped to the short arm of chromosome 7 (7p21) and composed of 6 exons with only 5 are coding (19, 20). Although, anti-inflammatory properties of this Th-2 type cytokine are well recognized, the role of IL-6 in pregnancy outcome remains unclear (21). Increased levels of this mediator have been associated with pregnancy complications such as pre-term birth (22). Several polymorphisms in IL-6 gene have been reported, some of which have been suggested to regulate its expression (23). Single nucleotide polymorphism (SNP) at position -634 C/G in the promoter region of the IL-6 gene is known to cause an altered promoter activity and thus resulting in a decreased production and secretion of IL-6 by peripheral blood mono-nuclear cells in vitro (24). 

In addition to identified causes of miscarriage, other factors increasing the risk of miscarriage have been reported. One of the attractive risk factors is the direct relationship between the number of live born children and pregnancy failure. Considering the potential role of cytokines in unexplained RPL, the objective of this study was analyzing the potential association between IL-6 634C/G polymorphism (rs 1800796) for the first time in Iran.

## Materials and methods

**Study population**

This case-control study has been performed in Arsanjan Islamic Azad University at Department of Genetics from August 2013 to October 2014. The cases comprise 121 women (17-85 years) with the history of at least two miscarriages of unknown reasons. To rule out any possible causes influencing the recurrent miscarriage, each woman underwent a diagnostic procedure includes hysteroscopy to detect uterine abnormalities, cell culture for Chlamydia and Mycoplasma infectious, maternal and paternal karyotyping, profiling hormonal status and asking for endocrine disorders. 

Depending on the history of previous pregnancies, women were divided to primary and secondary groups including; women with no successful pregnancy to term and those with a history of at least one pregnancy ending with live birth, respectively. 121 healthy postmenopausal women (43-76 years), were randomized to serve as a control group. No one has a history of miscarriage. 

All subjects in this study were from Fars province and match by geographic area. Written informed consent was obtained from all participants of the study before collecting blood samples. A questionnaire was used to elicit detailed information on demographic variables, the number of miscarriages, and number of children.

**Genotyping of IL-6 -634 C/G polymorphism**

Peripheral blood samples were collected in sterile tubes containing EDTA and genomic DNA was isolated using standard phenol- chloroform salting out method. -634C/G genotyping was done using PCR-RFLP method. The PCR primers were: 5´-GAGACGCCTTGAAGTAACTG-3´ (F) and 5´-AACCAAAGATGTTCTGAACTGA-3´ (R). PCR assay was performed for each sample in a final reaction volume of 25 µL, using 1 µL genomic DNA, 12.5 µL universal master mix (Ampliqon, Denmark), 1 µL forward primer, 1 µL reverse primer, together with 9.5 µL distilled water (DW). 

**Figure 1:**
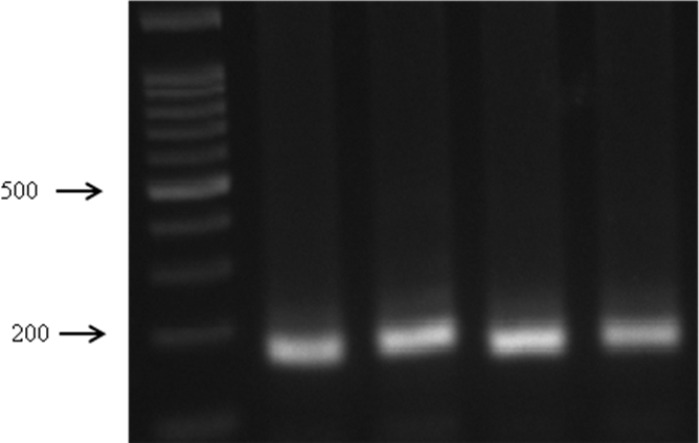
PCR amplification of the -634*C*/*G* polymorphism in the promoter of *IL-6* gene. using designed primer set produce 180 bp amplicon in length.

The PCR condition was as follows: Initial denaturation at 95^o^C for 3 min, followed by 30 cycles of: denaturation at 95^o^C for 30 Sec, annealing at 56^o^C for 30 and extension at 72^o^C for 30. Then one cycle of final extension step at 72^o^C for 5 min. All reactions were done using thermal cycler Applied Biosystems (Perkin Elmer 9600). The PCR products were digested with MbiI (BsrBI) restriction enzyme (Fermentas, # R0102S) and put at 37^o^C for 20 hr. The products were then resolved on 2% agarose gel electrophoresis and stain with syber gold DNA safe stain dye, then visualized using a UV transilluminator. DNA molecular weight marker (Fermentas, 100 bp Ladder) was used to assess the size of the PCR-RFLP products. The amplified fragment after digestion with MbiI restriction enzymes, gave rise to 2 fragments of 120 bp and 60 bp indicating the presence of polymorphic genotype (GG), the appearance of one undigested 180 bp fragment indicates the presence of homozygous wild type (CC), while the presence of 3 fragments at 180 bp, 120 bp and 60 bp indicates the heterozygous manner (CG) (Figure 1). 

**Statistical analysis**

A ^2^ test was performed for determining if the case and control groups’ demonstrate Hardy-Weinberg equilibrium. Statistical analysis was performed using the Statistical Package for Social Sciences (SPSS, version 16, Chicago, IL, USA). Numerical data of the scores were expressed as mean and standard deviation or median and range as appropriate. Qualitative data were expressed as frequency and percentage. Logistic regression was used to examine the relation between qualitative variables. Odds ratio (OR) with its 95% confidence interval (CI) was used for risk estimation. For quantitative data and comparison between two groups of case and control independent sample t-test was used. A p-value< 0.05 was considered significant. 

## Results

**Figure 2:**
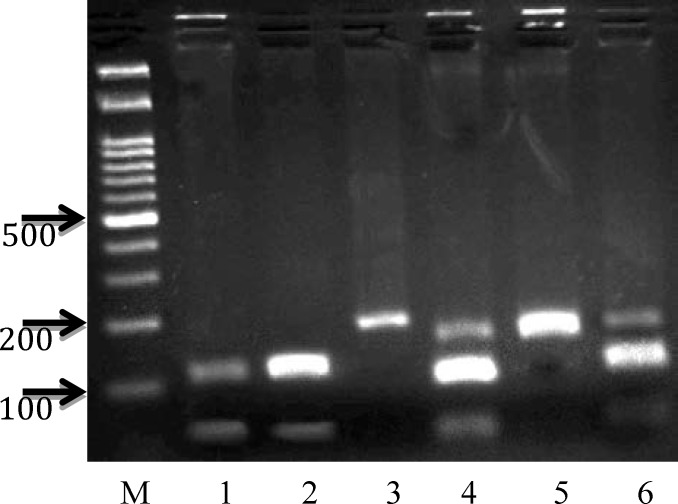
PCR-base restriction analysis of IL-6-634C/G polymorphism was shown on 2% agarose electrophoresis. The polymorphic region was amplified by PCR resulting in digestible fragment length 120 and 60 bp in lane 1, 2 (GG homozygote), indigestible fragment length 180 bp in lane 3 and 5 (CC homozygote). Three different fragments 180, 120, 60 bp (lane 4 and 6) represent CG heterozygote. M: marker, 100 bp ladder (Fermentas, Germany). [magnify: 640 × 480px

Results of PCR amplification and enzyme digestion were illustrated in figure 1 and 2 respectively. 121 women with RPL (35.6±12.5 years) and 121 healthy postmenopausal women (57.6±6.2 years) were analyzed for association. Studied subjects' characteristics are shown in table I. The mean number of miscarriages in patients was ranging between 2 - 10, which 23 women have 2 miscarriages and the remainders have 3 or more. The number of live births in the RPL group (1.3±2.2) was significantly lower than those in controls (4.8±2.3). This difference was significant (p<0.001). Genotypes and allele frequencies of women with RPL and controls are given in table II. 

The frequency of polymorphic allele G in patients with RPL was 75%, while in controls was 66% (p=0.036). This allele shows an abortive effect (OR=1.5, 95%CI: 1.03-2.27). Homozygote genotype GG (OR=5.5, 95%CI: 1.05-29.3, p=0.04) and the heterozygote GC (OR=4.9, 95%CI: 0.95-24.9, p=0.05) after adjusting for age, were associated with increased risk of RPL. Under the dominant model for the allele G (GG+GC vs. CC), this allele increased the risk of disease for more than 5 times (OR=5.1, 95%CI: 1.04-25.3, p=0.04). The genotype frequencies for both patients (^2^=2.1, df= 1, p>0.05) and controls (^2^=1.01, df= 1, p>0.05) in the studied SNP were not significantly differ from those expected for populations in Hardy- Weinberg equilibrium. 

**Table I:** Selected characteristics of the study subjects and potential risk factors for RPL

**Variables**			**Cases**	**Controls**	**p-value**
Age (mean±SD)			35.6 ± 12.5	57.6 ± 6.2	
Range			17-85	43-76	
Abortion (mean±SD)			3.3 ± 1.2	-	
No. abortion (n, %)					
		2	23 (19%)	-	
		≥3	98 (81%)	-	
Abortion type (n, %)					
	Primary		67 (55.4%)	-	
	Secondary		54 (44.6%)	-	
No. of live births			1.3 ± 2.2	4.8±2.3	<0.001

P<0.05 were considered to indicate statistical significance. (n=121)

**Table II:** Distribution of -634 C/G genotypes and allelic frequencies in cases and controls (Gene IL-6

**Genotype (n=121)**		**Controls (n, %)**			**Patients (n, %)**			a ** p-value**			**OR**			**95%CI**		
Genotype																
	CC			18 (14)			5 (4.1)			-			-			-		
	CG			47 (39)			50 (41.4)			0.05			4.9			0.95-24.9		
	GG			56 (47)			66 (54.5)			0.04			5.5			1.05-29.3		
Allele																
	C	83 (34)			60 (25)			-			-			-		
	G	159 (66)			182 (75)			0.036			1.5			1.03-2.27		
Phenotype																
	GG+GC vs. CC		103 (86)			116 (95.9)			0.04			5.1			1.04-25.3		
	CC+GC vs. GG		65 (53)			55 (45.5)			0.68			0.85			0.39-1.85		

OR: Odds ratio

CI: confidence interval

a adjusted for age.

## Discussion

Although extensive studies have been made on the role of several cytokines, including IL-6, IL-10, TNF-α and IFN-γ in different stage of pregnancy, the inconclusive results, make the interpretation of their effect on pregnancy difficult (16, 25, 26). One of the well-studied cytokine is IL-6, which the reduction of its protein and mRNA in the mid- secretary phase of endometrial tissue in prone women to RPL has been shown in distinct studies (27). As compared with normal women, patients with the history of RPL, have significantly lower serum concentrations of Th-2 cytokines including IL-6 and IL-10 (28). The production of pro-inflammatory and anti-inflammatory cytokines is partly under genetic control. In present research emphasis is laid on IL-6 gene polymorphism as this cytokine supposed to have crucial role on the implanting embryo. One of the most common polymorphism in the promoter region of the IL-6 gene is 174 G/C, known to alter the IL-6 transcription, which its G allele shows relation to increase serum concentrations only in one study and with decreased in others (29, 30). 

The literature review demonstrates that the vast majority of the studies assessed the role of IL-6 in pathogenesis of RPL, focused on this SNP, which none of them resulted in any association (26, 28, 31). Several studies on the association of the different cytokine gene polymorphisms and RPL were published in Iran. In one study considering the role of -174 C/G (IL-6), -197G/A (IL-17) and [-592A/C, -819C/T, -1082A/G (IL-10)] in the pathogenesis of the RPL, no significant differences was found between RPL cases and controls (32). In the study by Nematollahi *et al* the effectiveness of the -964 A>G polymorphism in IL-27 gene in the RPL etiology was evaluated. They found no significant relationship between this polymorphism and the occurrence of the RPL in Iranian women (33). 

The results of the association assessment between the polymorphisms of the IL-17A (rs2275913) and the IL-17F (rs 763780) genes with the RPL in Iranian women suggested the IL-17 F as a risk factor for RPL (34). Daher *et al* reviewed all five available studies in Europe to explore the role of IL-6 174 polymorphism on the RPL incidence (24). No differences between RPL patients and controls were detected concerning IL-6 genotype frequencies in any of the studies, even in the Japanese population not a single polymorphic allele was detected neither in patients nor in the control group (24). Since the results influenced strongly by ethnicity, they were not comprehensive and could not apply to all populations. The Caucasian Northern Ireland group is the only one population with a lower IL-6 174 G allele frequency relative to other populations (35). 

As compared to the Japanese population, in the study of women from Africa, complete absence of homozygous CC genotype was seen (36). The other common regulatory polymorphism in the promoter of the gene encoding for IL-6 suggested to affect the expression of the gene is cytosine (C) to guanine (G) substitution -634 base pair downstream of the start point. The only study assessed the association between -634 C/G polymorphism and RPL was in Japan, which is resulted in the significant differences in genotype frequencies (CC vs. GG+GC) between women with recurrent pregnancy loss and the control group. Statistical analysis shows the protective role of the carriers of G allele in RPL susceptibility (OR=0.46, 95% CI:0.24-0.91) (31). 

To our knowledge, this is the first study of the association between IL-6 -634 C/G polymorphism and RPL in Iran, and the second one after the data published in Japan. Based on the anti-inflammatory role of the IL- 6 in reproduction and due to lower IL-6 expression, the polymorphic GG genotype might be observed in the women with RPL. This assumption confirmed by the results showing the higher frequency of GG+GC vs. CC genotypes among patients in comparison to control group (p=0.04, OR=5.1, 95%CI: 1.04-25.3). Therefore, the G allele of this polymorphic site could be considered as a risk factor for RPL among women from Iran. Whether the G allele of this polymorphic site reduces the IL-6 gene expression or not should be studied later. Our results are inconsistent with data published from the Japanese experiment, as they introduce the G allele as a protective factor against RPL (OR=0.46) (31). 

The number of live births among women with the history of RPL was significantly lower than healthy women (p<0.001). This data also highlights the effectiveness and the crucial role of normal IL-6 expression in the survival of pregnancy outcome, which reduced at the presence of mutant allele G of -634 SNP. 

## Conclusion

In summary, the present study suggested that polymorphism of the IL-6 gene (-634C/G) is associated with increased risk for RPL in Iranian women. Considering that the IL-6 GG genotype is associated with increased risk for RPL in Iran but not in Japanese population, most epidemiologic studies are necessary to further ascertain the relationship between IL-6 promoter polymorphism (-634C/G) and genetic predisposition to RPL (31). 
